# ΔNp63 is a pioneer factor that binds inaccessible chromatin and elicits chromatin remodeling

**DOI:** 10.1186/s13072-021-00394-8

**Published:** 2021-04-17

**Authors:** Xinyang Yu, Prashant K. Singh, Shamira Tabrejee, Satrajit Sinha, Michael J. Buck

**Affiliations:** 1 Department of Biochemistry, State University of New York at Buffalo, Buffalo, NY 14203 USA; 2 Department of Biomedical Informatics, Jacobs School of Medicine & Biomedical Sciences, Buffalo, USA; 3 Present Address: Zhuhai Interventional Medical Center, Zhuhai Precision Medical Center, Zhuhai People’s Hospital, Zhuhai Hospital Affiliated with Jinan University, Zhuhai, Guangdong China

**Keywords:** P63, Pioneer factor, Nucleosome, Chromatin modification

## Abstract

**Background:**

ΔNp63 is a master transcriptional regulator playing critical roles in epidermal development and other cellular processes. Recent studies suggest that ΔNp63 functions as a pioneer factor that can target its binding sites within inaccessible chromatin and induce chromatin remodeling.

**Methods:**

In order to examine if ΔNp63 can bind to inaccessible chromatin and to determine if specific histone modifications are required for binding, we induced ΔNp63 expression in two p63-naïve cell lines. ΔNp63 binding was then examined by ChIP-seq and the chromatin at ΔNp63 targets sites was examined before and after binding. Further analysis with competitive nucleosome binding assays was used to determine how ΔNp63 directly interacts with nucleosomes.

**Results:**

Our results show that before ΔNp63 binding, targeted sites lack histone modifications, indicating ΔNp63’s capability to bind at unmodified chromatin. Moreover, the majority of the sites that are bound by ectopic ΔNp63 expression exist in an inaccessible state. Once bound, ΔNp63 induces acetylation of the histone and the repositioning of nucleosomes at its binding sites. Further analysis with competitive nucleosome binding assays reveal that ΔNp63 can bind directly to nucleosome edges with significant binding inhibition occurring within 50 bp of the nucleosome dyad.

**Conclusion:**

Overall, our results demonstrate that ΔNp63 is a pioneer factor that binds nucleosome edges at inaccessible and unmodified chromatin sites and induces histone acetylation and nucleosome repositioning.

**Supplementary Information:**

The online version contains supplementary material available at 10.1186/s13072-021-00394-8.

## Introduction

Gene regulation is controlled by transcription factors (TFs), which switch genes on and off in a spatial and temporal manner. TFs are sequence-specific DNA-binding proteins that recognize and bind to evolutionally conserved but often degenerative DNA sequences known as TF motifs [1, 2]. These degenerative TF motifs can occur millions of times across the human genome, but only a small proportion of these sites are actual bound in vivo—the mechanisms of how this targeted TF binding takes place remains ill-understood [3].

The transcription factor p63 plays a pivotal role in maintaining epidermal lineage and in the epidermal commitment steps during skin development [4, 5]. The critical role of p63 in epidermal morphogenesis is evident from the distinct phenotype of p63-null mice that includes defects of limbs, craniofacial region, total loss of squamous epithelia and agenesis of other epithelial-rich tissues [6, 7]. *TP63* mutations in human also cause severe developmental diseases, such as ectrodactyly ectodermal dysplasia–clefting syndrome (EEC), limb–mammary syndrome (LMS), ankyloblepharon and ectodermal dysplasia–clefting syndrome (AEC), split-hand/foot malformations (SHFM) and Rapp–Hodgkin syndrome [8]. p63 plays a versatile role as a master regulatory factor and affects a myriad of cellular processes, from basement membrane formation, barrier formation, terminal differentiation, to epithelial cell adhesion and proliferation. In humans and mice, p63 protein has multiple isoforms due to alternative mRNA splicing and usage of different promoters [9–11]. The ΔNp63α isoform is the most predominant in epithelial cells and possess the majority of p63 biological functions [8, 12–14].

While the role of TP63 in regulating epidermal morphogenesis and other epithelial-rich tissues have been extensively investigated, the mechanism of how ΔNp63 modulates chromatin still remains elusive. ΔNp63 has been proposed to be both an activator and a repressor of transcription. As an example while ΔNp63 can activate important epidermal genes such as the keratin genes K5 and K14 [14, 15], it can also act to repress expression of genes for non-epidermal lineages [16]. In its repressive role ΔNp63 has been demonstrated to physically interact with the histone deacetylase HDAC1 and HDAC2 [17]. Conversely, the Swi/Snf chromatin remodeling complex, BAF has been shown to maintain nucleosome displacement at ΔNp63 binding sites [18]. Furthermore, a recent study has found an interaction between H3K4 histone methyl-transferase, KMT2D, and ΔNp63 in epidermal keratinocytes [19]. These studies suggest that one potential function of DNA-bound ΔNp63 could be to remodel the neighboring chromatin, thereby creating an active or repressive chromatin state.

Our previous studies have revealed that ΔNp63-bound sites across the genome in human keratinocytes are associated with an accessible and active chromatin environment [20]. However, we found that those sequences were predicted to have a higher chance of nucleosome deposition, suggesting that the accessible chromatin landscape at ΔNp63-bound sites might be driven by the pioneering activity of ΔNp63 [20]. To directly test if ΔNp63 is a pioneer factor capable of binding inaccessible chromatin and remodeling the neighboring chromatin, here we have performed studies with ectopically expressed ΔNp63 in cell lines that are ΔNp63 naïve. This has allowed us to directly determine the global state of nucleosome modifications and chromatin accessibility required for ΔNp63 binding and the consequences of binding. By combining our analysis with in vitro nucleosome binding assays, we demonstrate that ΔNp63 is a pioneer factor capable of binding directly to nucleosome edges at chromatin inaccessible regions in a histone modification-independent manner.

## Results

Our previous examination of ΔNp63 binding in NHEK (normal human epidermal keratinocytes) cells demonstrated an active chromatin signature at many of its binding sites consisting of high H3K27ac, H3K9ac, H3K4me1, H3K4me2, and H3K4me3 along with increased chromatin accessibility [20]. These sites while depleted of nucleosomes in NHEK contained nucleosome-preferring sequences. However, it was unclear from this analysis if ΔNp63α played an active role in establishing this chromatin architecture or required an a priori permissive chromatin environment to bind. Therefore, to directly test ΔNp63 pioneering capabilities, we established an inducible system in which ΔNp63 is ectopically expressed in a p63-naïve cell line, K562. K562 is a widely used immortalized myelogenous leukemia cell line and is an advantageous choice for these experiments because no p63-isoforms are expressed and there are extensive genomic and epigenomic datasets available from the ENCODE project [21]. Two doxycycline (Dox) inducible cell lines were generated containing the wild-type (WT) HA-tagged ΔNp63α and DNA-binding mutant ΔNp63α(R304W). Western blot analysis showed that both WT and mutant ΔNp63α were expressed after Dox induction and the levels comparable to those observed in epithelial cells (Additional file 1: Figure S1).

ΔNp63α (WT) and ΔNp63α(R304W) were induced in K562 cells with Dox and ChIP-seq experiments performed on two biological replicates with ΔNp63-specific and anti-HA antibodies. ChIP-seq experiments with anti-p63 4A4 antibodies identified 2049 sites while the HA antibody identified 10,900 sites. The differences in peak numbers is likely due to differences in antibodies specificity. In contrast, ChIP-seq experiments for ΔNp63α(R304W) show limited enrichment consisting of only 29 sites for 4A4 antibody and 68 sites for HA antibody with zero overlapping sites. Both ChIP-seq experiments for ΔNp63α enriched for DNA sequences containing the p63 binding motif (Additional file 1: Figure S2). Representative genomic locations showing both wild type and mutant ΔNp63α ChIP-seq further demonstrate the lack of signal for the ΔNp63α(R304W) ChIP experiments (Additional file 1: Figure S3). In total 1980 common sites that were identified in experimental replicates using WT ΔNp63α ChIP but not in the control cells expressing ΔNp63α(R304W) were chosen for further analysis.

**Fig. 1:**
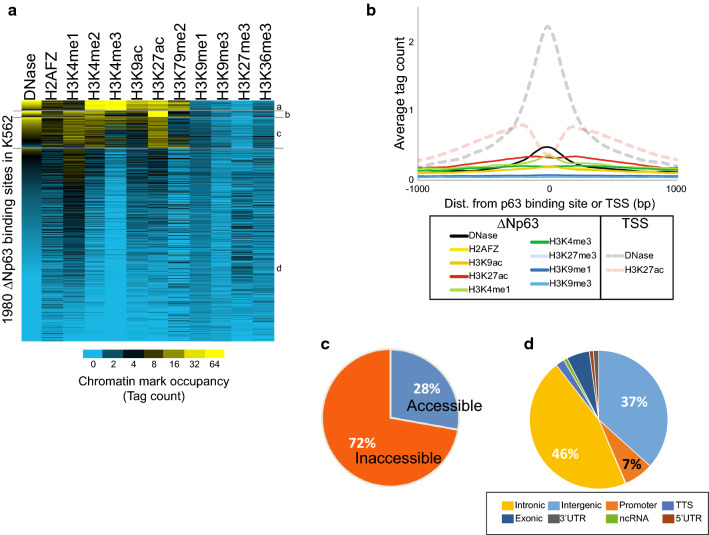
ΔNp63 binds inaccessible unmodified chromatin in K562. **a** 1980 high-confidence ΔNp63 bound sites in K562 were Kmeans clustered into 4 groups by DNase, H2AFZ, 5 active (H3K4me1, H3K4me2, H3K4me3, H3K9ac, H3K27ac), 2 repressive (H3K9me3, H3K27me3) and other (H3K79me2, H3K9me1, H3K36me3). Chromatin datasets are from K562 in the absence of ΔNp63 from the ENCODE project. **b** Average chromatin architecture in K562 at 1980 ΔNp63 ChIP-seq summits compared to 72,291 transcriptional start sites (TSS). Data are plotted for 2 kb flanking the summit or TSS. **c** Chromatin accessibility for the 1980 ΔNp63-bound sites in K562. Accessibility is defined from the synthesis track of DNase and FAIRE from ENCODE (GSM1002657) [66]. **d** Genomic annotation for the ΔNp63-bound sites in K562

To understand the chromatin characteristics required for ΔNp63α binding, we examined the robust dataset of chromatin modification marks and accessibility for K562 that have been generated by the ENCODE project. This provided us with the chromatin characteristics before ΔNp63 was induced in our experiments. Ten histone modifications H3K4me1, H3K4me2, H3K4me3, H3K9ac, H3K9me1, H3K9me3, H3K27ac, H3K27me3, H3K36me3, H3K79me2 with DNase-seq and H2AFZ profiles were examined and clustered into 4 groups (Fig. 1a). Groups a, b, c contain a total of 374 sites and appear to represent binding locations occurring in already active chromatin environments with high levels of H3K27ac and H3K4me1 or H3K4me3. The majority of the ΔNp63α-bound sites (1606 out of 1980) are in group d and represented genomic segments that were bereft of signal for any histone modification tested. The average chromatin architecture further demonstrates the absence of histone modifications and is shown in comparison to the active chromatin state at transcriptional start sites (TSS; Fig. 1b). Examination of chromatin accessibility by DNase-seq shows that the majority of these (1429 of 1980) regions are located at inaccessible sites in K562 as defined by DNase-seq (Fig. 1c). These sites are located predominantly in intronic (46%) and intergenic (37%) regions of the genome similar to ΔNp63α binding in NHEK cells where it targets 47% intronic and 39% intergenic regions (Fig. 1d) [22].

**Fig. 2:**
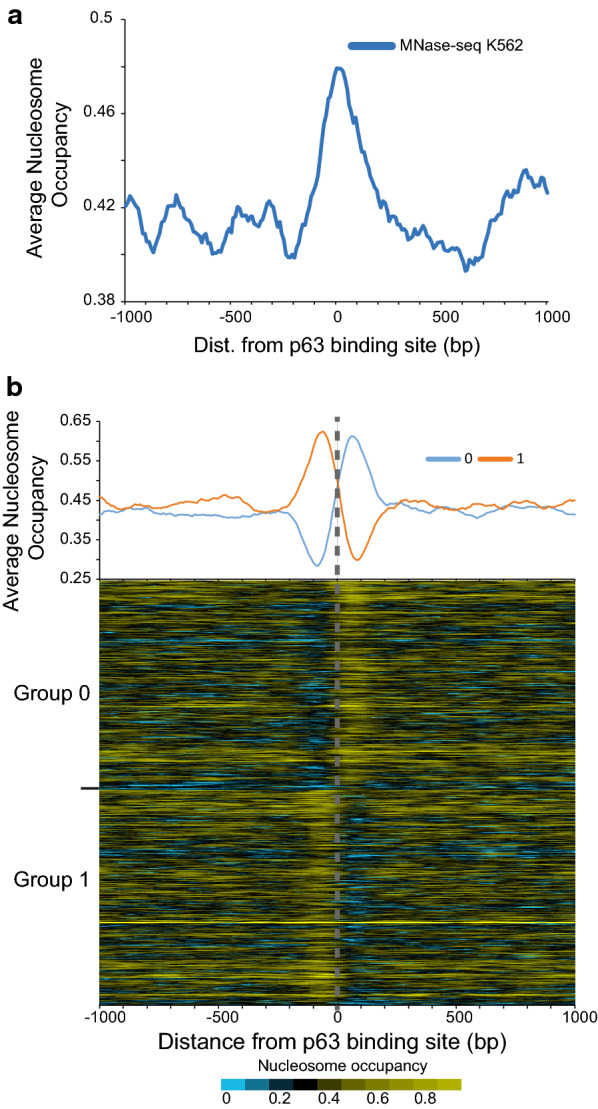
Nucleosome occupancy is enriched at ΔNp63 binding sites. **a** Average nucleosome occupancy at ΔNp63 ChIP-seq peaks centered on p63BS determined from MNase-seq Mieczkowski et al. [50]. **b** Nucleosome occupancy at each ΔNp63 binding site with the sites grouped by symmetry around the p63BS. Standardized and extended MNase-seq reads were clustered by into two group [23]. Average nucleosome occupancy for each group is plotted on top with a heat map showing individual sites below

To further characterize the chromatin before ΔNp63α binds, we examined nucleosome occupancy as determined from MNase-seq. Nucleosome occupancy and nucleosome positions from two independent MNase-seq datasets show nucleosome enrichment at these binding sites before ΔNp63α binds in K562 cells (Fig. 2a and Additional file 1: Figure S4). This peak of signal can be caused by the 2 closely flanking nucleosomes with binding taking place at the edges. To address this further, we examined nucleosome occupancy at p63BS after grouping bound locations by the nucleosome symmetry at the site (Fig. 2b) [23]. Results show ΔNp63α binding occurring 50–60 bp from the peak of nucleosome occupancy.

### Comparison of ΔNp63 binding sites between NHEK and ectopic-induced binding in K562

**Fig. 3:**
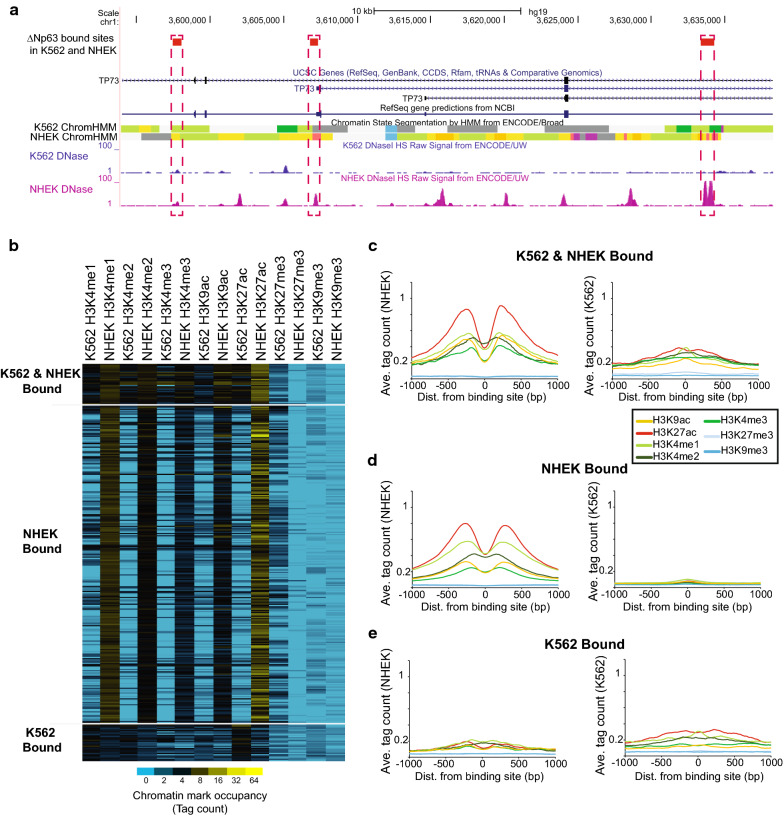
Chromatin at ΔNp63 binding sites in K562 and NHEK. **a** UCSC Genome Browser image showing ΔNp63 binding sites shared in both NHEK and K562 near the TP73 gene. Chromatin State Segmentation (ChromHMM) and DNase data are plotted for both K562 and NHEK. Chromatin states are defined by the composite track of Broad ChromHMM from ENCODE (GEO GSE38163 GSM936088, GEO GSE38163 GSM936087) [66]. Fifteen HMM states are associated with different segment colors: state 1-bright red-active promoter, state 2-light red-weak promoter, state 3-purple-inactive/poised promoter, state 4-orange-strong enhancer, state 5-orange-strong enhancer, state 6-yellow-weak/poised enhancer, state 7-yellow-weak/poised enhancer, state 8-blue-insulator, state 9-dark green-transcriptional transition, state 10-dark green-transcriptional elongation, state 11-light green-weak transcribed, state 12-Gy-polycomb repressed, state 13-light gray-heterochromatin; low signal, state 14-light gray-repetitive/copy number variation, state 15-light gray-repetitive/copy number variation. **b** ΔNp63 sites bound in K562 and NHEK are grouped into K562 & NHEK (1037 sites), NHEK only (8195 sites), and K562 only (943 sites). Chromatin datasets are from K562 and NHEK from the ENCODE project. Average chromatin architecture in the specified cell line for ΔNp63 bound site in **c** both K562 and NHEK, **d** NHEK only, or **e** K562 only

To further examine the chromatin characteristics at ΔNp63α binding sites, we compared the ΔNp63α-bound K562 targets to the binding sites in a normal ΔNp63α expressing cell type NHEK [20, 22]. Sites were classified as jointly bound, or bound only in each specific cell type. The consequence of ΔNp63 binding can be discerned by comparing the chromatin at sites bound in a ΔNp63 expressing cell line, NHEK, with the chromatin seen at the bound sites in K562. Three binding sites near the TP73 gene exemplify the chromatin differences in the two cell lines (Fig. 3a). The sites bound in K562 are often within heterochromatin chromatin states (gray) or weak-TX (green). In NHEK, these same sites are shown to be active as strong enhancers (red) or weak enhancers (yellow). The DNase signal is also higher in 2 of 3 binding sites in NHEK.

Sites bound by ΔNp63 in both cell lines show active histone modifications in NHEK and the relative absence of similar modifications in K562 (Fig. 3b, c). Sites in NHEK are flanked by transcriptional active histone modifications (H3K9ac, H3K27ac, H3K4me1, H3K4me2, H3K4me3), while in K562 these same sites show reduced signal. Sites bound only in NHEK (Fig. 2b middle, d) have high levels of active histone modifications, while all modifications display low signal in K562. K562-specific sites (Fig. 3b bottom, e) display relatively low levels of active histone modifications in both NHEK and K562.

Examination of ΔNp63-sites bound only in NHEK allows us to address the role of repressive histone modifications (H3K27me3 and H3K9me3). While these 8195 NHEK-exclusive sites represent bona fide targets of ΔNp63, they were not occupied by ectopically expressed ΔNp63 in K562. The observation that these sites are not enriched for repressive histone modifications suggest that active repression might not be a strong driving force in preventing or blocking ΔNp63 binding to these sites in vivo.

### ΔNp63α can bind inaccessible, inactive sites in HepG2

**Fig. 4:**
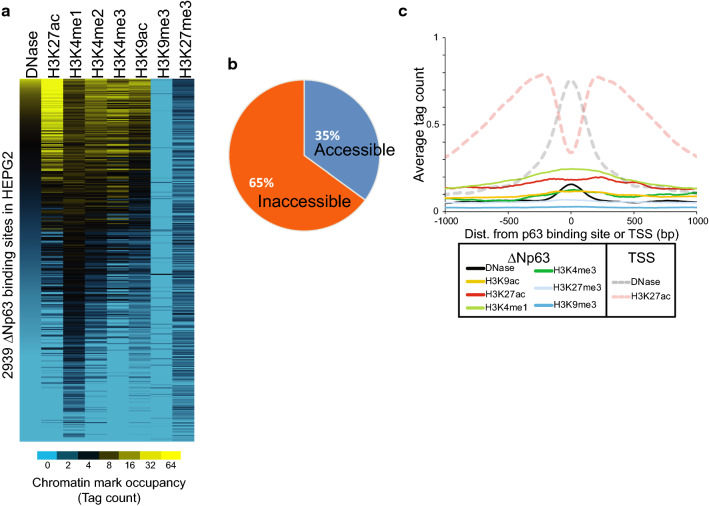
ΔNp63 expression in HepG2 leads to binding at inaccessible and unmodified chromatin regions. **a** The 2939 ΔNp63-bound sites in HepG2 cells with their histone modifications and DNase measurements. Sites are sorted by DNase signal. Chromatin datasets are from HepG2 in the absence of ΔNp63 from the ENCODE project. **b** Chromatin accessibility for the 2939 ΔNp63-bound sites in HepG2. Accessibility is defined from the synthesis track of DNase and FAIRE from ENCODE (GSM1002654) [66]. **c** Average chromatin architecture in HepG2 at inaccessible ΔNp63 ChIP-seq summits compared to transcriptional start sites (TSS). Data are plotted for 2 kb flanking the summit or TSS

To validate our results from K562 cell line-based studies and to examine if ΔNp63α can also target inactive and inaccessible chromatin in other cell types, we developed a ΔNp63α -expressing HepG2 cell line. HepG2 is p63-naïve and is a widely used human cell line derived from hepatic cancer that has been extensively characterized for studies of the endoderm linage. By performing ChIP-seq experiments, we identified ΔNp63-bound 2939 targets in HepG2 (Additional file 1: Figure S5). Most sites bound in HepG2 have low levels of histone modifications and accessibility, which is consistent with what was observed in K562 (Fig. 4a). In addition, the majority, 65%, are classified as inaccessible (Fig. 4b). The chromatin architecture was further examined at the inaccessible bound sites and displayed a low signal compared with the chromatin modifications at TSS (Fig. 4c). Taken together, our studies with ectopic ΔNp63α expression in two independent cell lines reaffirmed the notion that ΔNp63α can bind inaccessible sites in the genome.

### ΔNp63 binds at nucleosomes and leads to H3K27ac and nucleosome depletion

**Fig. 5:**
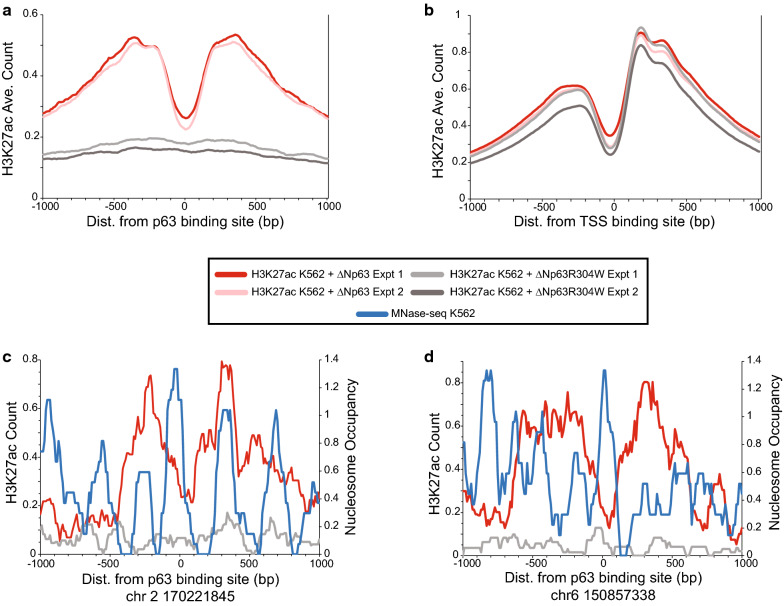
ΔNp63 binding causes H3K27ac of flanking nucleosomes. **a** Average chromatin architecture for H3K27ac at ΔNp63 ChIP-seq peaks centered on p63BS. Data are plotted for each H3K27ac ChIP-seq experiment from K562 + ΔNp63 and K562 + ΔNp63(R304W) control experiments. **b** H3K27ac at transcriptional start sites (TSS). **c**, **d** H3K27ac at two p63BS. Average H3K27ac from two replicate experiments for K562 + ΔNp63 and K562 + ΔNp63(R304W). Nucleosome occupancy as determined from MNase-seq from non-p63 expressing K562 [50]

Our comparison between K562 and NHEK binding sites suggest that ΔNp63α binding leads to an active nucleosome architecture. To test the consequences of direct effects of ΔNp63α binding, we determined if H3K27ac increased at sites bound by ΔNp63α. ChIP-seq for H3K27ac was performed in ΔNp63α and ΔNp63α(R304W) expressing K562 cell lines (Fig. 5). We found that binding of ΔNp63α leads to an increased in H3K27ac at ΔNp63α-bound sites while induction of DNA-binding mutant ΔNp63α(R304W) does not change the H3K27ac (Fig. 5a). By comparison, H3K27ac does not change at TSS in K562 in either cell line (Fig. 5b). In addition, these results show the characteristic peak–valley–peak for the H3K27ac surrounding the ΔNp63 binding sites, suggestive of nucleosome depletion at the binding site. Examination of single sites further highlights the starting structure and consequences of ΔNp63α (Fig. 5c, d; Additional file 1: Fig. S7). At these sites the p63BS is located within a well-positioned nucleosome. After induction of ΔNp63α the flanking nucleosomes are acetylated with a dip in signal at the site of binding, suggesting remodeling of the centrally located nucleosome.

### ΔNp63 binds nucleosome edges

**Fig. 6:**
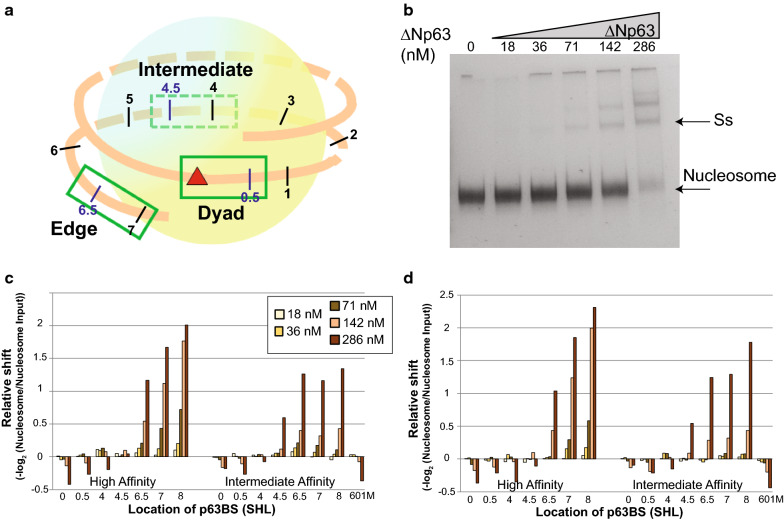
ΔNp63 binds to nucleosome edges. **a** A 217-bp dsDNA library was designed containing p63BS in various nucleosomal positions. A 20-bp-long p63BS (intermediate and high affinity) was placed at different positions 0, 5, 41, 46, 66, 71, and 81 bp away from the dyad. The superhelix location (SHL) is designated for each nucleosome sequence. Within the nucleosome, three different positions (dyad, intermediate, and edge) were chosen with increasing distance to nucleosome dyad. **b** Nucleosomes containing 16 different sequences were bound to increasing amounts of ΔNp63 and separated by native PAGE. Lanes contain the following: 1–0.25 pmol nucleosomes, 2–5 contain 0.25 pmol of nucleosomes with 18, 36, 71, 142, or 286 nM of ΔNp63 (0.125, 0.25, 0.5, 1, or 2 pmol). Nucleosome and the supershift (Ss) bands are indicated. **c**, **d** Relative shift for each nucleosome is determined by counting the frequency of each sequence within the nucleosome band and comparing it to non-specific binding to the 601 sequence. This value is then normalized to the input ratios of nucleosomes. The alternative control sequence 601-modified (601 M) is plotted. Results for each experimental replication are plotted in **c**, **d**

To further understand the ability of ΔNp63 to bind to inaccessible chromatin, we tested ΔNp63 binding to nucleosome DNA with a competitive nucleosome binding assay that we have recently developed [24]. We generated 16 templates derived from Widom 601 nucleosome positioning sequence, of these 14 nucleosome templates contained a high-affinity or intermediate-affinity p63 binding site (p63BS) that were placed at increasing distance to the nucleosome dyad axis, in an exposed or concealed rotational orientation (Fig. 6a; Additional file 1: Fig. S8A). Two nucleosome sequences lacking p63BS served as internal control (Additional file 2: Table S1). Nucleosome population were obtained after in vitro reconstitution with unmodified histones via salt gradient dialysis on all nucleosome sequences simultaneously, and purified from free DNA with a sucrose gradient.

ΔNp63 was added to 0.25 pmol purified nucleosome at increasing concentration (0 to 2 pmol, 0 to 286 nM). The binding reactions were then separated on a native polyacrylamide gel to detect the ΔNp63–nucleosome complex (Fig. 6b). The first lane contained only nucleosomes and was used to measure background and input levels for each experimental replicate. As ΔNp63 concentration increased a supershifted band appears and intensifies at higher amounts of ΔNp63. As the concentration of ΔNp63 was increased, additional supershifts were observed while the nucleosome-only band intensity significantly decreased, similar to our published findings for p53 [24]. The multiple supershifts are likely due to ΔNp63 dimers and tetramers binding p63 half-sites and full-sites, respectively [25].

DNA was then extracted and purified from the supershifted and nucleosome band and sequenced. The sequencing results were then mapped back to the original 16 nucleosome sequences and compared to the control non-specific sequence in the same lane. This analysis method controls for non-specific binding, gel loading, PCR amplification, and next-generation sequencing. This approach is performed on each non-shifted nucleosome band independently to see what types of nucleosomes are bound by ΔNp63 and shifted, two replicates were conducted (Fig. 6c, d). Our analysis demonstrates that nucleosomes containing a high-affinity or intermediate-affinity p63BS located around the nucleosomes boundaries at superhelix location (SHL) 6.5, 7, and in the linker SHL 8 are bound first at the lowest concentrations. In contrast, the nucleosomes containing a p63BS located near the dyad are not specifically bound as compared to the control nucleosomes. Examination of the supershifted fragments show similar results (Additional file 1: Figure S8B, C), and is consistent with ΔNp63 binding only at nucleosome edges.

## Discussion

Several studies have examined the chromatin environment at TF binding sites [26, 27]. These studies often cannot address cause and effect due to the fact that the binding of TF is often examined concurrently in specific cell lines with experiments that identify the prevailing global chromatin environment. In our previous studies on ΔNp63 binding patterns in human and mouse keratinocytes, we found that ΔNp63 binding occurred at predominately active chromatin regions containing active histone modifications (H3K27ac, H3K4me1, and H3K4me3) [20, 28]. In human keratinocytes two pieces of evidence suggested that ΔNp63 was pivotal in potentially creating the chromatin environment. First, ΔNp63-bound sites have higher sequence-defined nucleosome occupancy, suggestive of a positioned nucleosome over the binding site in other cell types. Second, at chromatin open/accessible locations there was a further positive association between DNase signal and the quality of the p63BS [20]. These finding suggested that ΔNp63 might function as pioneer factor that can bind directly to nucleosomes and regulate epithelial differentiation.

To directly test ΔNp63 pioneer ability, we used in vivo and an in vitro assays to determine the chromatin environment at ΔNp63 binding sites before ΔNp63 is present, and the ΔNp63 ability to bind directly to nucleosomal DNA. K562 and HepG2 cells do not express any of the known p63-isoforms making their chromatin p63-naïve. Thus, we reasoned, by expressing ectopic ΔNp63 and locating its binding sites by ChIP-seq, we can determine the chromatin environment conducive for ΔNp63 binding. In both cells lines, we found ΔNp63 is able to bind its binding sites located in inaccessible chromatin without the active histone modifications (H3K27ac, H3K4me1, H3K4me3, H3K9ac). Our results are consistent with recent studies in Zebrafish where p63 was shown to bind to non-accessible chromatin at epidermal enhancers during vertebrate development [29]. One limitation of our Dox induction experiments in K562 and HepG2 cell lines is that the expression level of ectopic ΔNp63α might vary across individual cells. This can cause skewing of results due high ΔNp63α expression only in a subpopulation of cells, future studies at a single-cell resolution will allow us to circumvent this problem.

To understand the mechanism for ΔNp63 binding to inaccessible chromatin we examined ΔNp63 binding to nucleosomal DNA with a competitive nucleosome binding assay [24]. In these experiments ΔNp63 can bind to its binding sites when they are located near the nucleosome edge and binding is inhibited within 50 bp of the nucleosome dyad. The results we obtained with ΔNp63 are similar to TP53, which also preferentially binds at nucleosome edges [24, 30]. Interestingly, our observations are consistent with a model in which the dynamic partial unwrapping near nucleosome edges where DNA near the entry–exit region is unwrapped from the histone proteins exposing the DNA to TF binding [31, 32]. We therefore posit that ΔNp63 can access the partially unwrapped nucleosome and remain stably bound.

To understand the consequences of ΔNp63 binding we examined the chromatin states at sites bound in both K562 and NHEK. In K562 cell line, we found that many of the sites that were bound by ΔNp63 were shown to be in repressive chromatin states. In NHEK, the same genomic regions are characterized by a more active chromatin state, suggesting a direct role for ΔNp63 in activating chromatin. To validate ΔNp63 ability to activate chromatin domains we found a strong increase in H3K27ac at flanking nucleosomes after ΔNp63 binding. This peak–valley–peak signal is characteristic of active regulatory regions and represents the formation of a nucleosome-depleted region occurring at the binding site. Recent studies expressing ΔNp63 in dermal fibroblasts also showed increased chromatin accessibility and H3K27ac at ΔNp63 binding sites [33]. ΔNp63 has been shown to require the BAF complex to maintain a nucleosome-depleted region at its binding sites [18], and has been shown to interact with H3K4 methyl-transferase, KMT2D [19]. These observations and our findings reported here suggest that ΔNp63 ability to remodeling chromatin after binding is not a cell type-specific function, and that ΔNp63 can directly recruit general chromatin remodeling co-activators.

Despite ΔNp63’s ability to act a pioneer factor that can bind to nucleosomal DNA and elicit chromatin remodeling, the underlying molecular mechanisms differs from other well-characterized pioneer factors. For example, the canonical pioneer factor FOXA [34, 35] can bind the nucleosome dyad and displace the linker histone to maintain nucleosome accessibility [36]. The Yamanaka factors SOX2 and OCT4 [37] can bind heterochromatin domains, though heterochromatin impedes their binding [38]. As more pioneer factors are characterized, it is becoming clear that they interact with nucleosomes and histone modifications in varied manner.

The results from our studies have some limitations on defining ΔNp63 targeting specificity. First, the experiments we employed with K562 and HepG2 used unsynchronized replicating cells. During DNA replication RNA synthesis is greatly reduced and there are major changes to chromosome architecture including breakdown of nuclear envelop, chromosome condensation, and loss of long range interactions between enhancers and promoters [39, 40]. In addition, many TFs are undetectable in mitotic chromosomes [41], while a subset of TF are retained and bound during mitosis [42]. These “book marking” TF enable the proper activation of gene expression after mitosis and include pioneer factors FoxA1, Sox2, Oct4, and Klf4 [42]. Notably, many of the K562 induced ΔNp63-binding sites are associated with enhancer regions, and other groups have proposed that ΔNp63 acts to ‘bookmark’ genes for expression in stratifying epithelia [33, 43–45]. A second limitation of our studies is that it does not provide a clear rationale for why only specific subset of genomic sites are bound by ΔNp63 and what might be the chromatin modifications that dictate such choices. In this regard, results from the p63–nucleosome in vitro binding assays shed some light. We show that ΔNp63 could only bind its target BS when it was near the nucleosome edge. In addition, examination of the ΔNp63 ChIP-seq sites shows a dual peak of nucleosome enrichment. These finding suggest that ΔNp63 binding specificity might be driven in part by the location of the p63BS within the nucleosome. Computational modeling of how ΔNp63 binding is inhibited within 50 bp of the dyad and de novo prediction of ΔNp63 binding when p63 is ectopically expressed, however remain un-conclusive suggesting that additional unexplored factors influence ΔNp63 binding site selection.

## Materials and methods

### Cell culture and treatment

Human K562 cell line was grown in Roswell Park Memorial Institute (RPMI) 1640 medium (Hyclone SH30027.LS) supplemented with 1% penicillin/streptomycin (Gibco 15140-163), 4 mM L-glutamine (Gibco 25030-164) and 10% fetal bovine serum (Giboco SH30071.03). HepG2 cells were grown in Dulbecco’s modified Eagle’s medium (DMEM) supplemented with 1% penicillin/streptomycin (Gibco) and 10% fetal bovine serum (Gibco). The identities of all cell lines were confirmed via STR profiling and cultures are routinely checked for mycoplasma contamination. All cell lines were incubated at 37 °C and 5% CO_2_. The cDNAs corresponding to human ΔNp63α and ΔNp63α(R304W) (ΔNp63α with an amino acid substitution in DBD thus losing specific sequence binding ability) were cloned into the pINDUCER21 vector, respectively. Stable cell lines expressing corresponding cDNAs were generated according to the pINDUCER lentivirus toolkit [46], which was followed by cell sorting to select the high-GFP population indicating stable expression of introduced ΔNp63α. FACsorting was performed on a BD Biosystems AriaII by Roswell Park Cancer Institute, Department of Flow and Image Cytometry. After cell selection, 300 ng/ml doxycycline were added individually to different cell lines after they were grown to > 60% confluency in order to induce p63 expression. Expression of ΔNp63α was validated by western blot with ΔNp63-specific in-house generated antibody, ΔNp63α original expressing epithelial cell line A253 was applied as positive control to confirm ΔNp63α ectopic expression in engineered cell lines.

### Chromatin immunoprecipitation (ChIP) and library preparation

Cell pellets were then collected after 18 h of Dox induction, and then fixed with 1% formaldehyde for 10 min, approximately 1 to 4 million cells were prepared for each ChIP-seq experiment. ChIP experiments were performed as described previously [47, 48], with sheared chromatin from 1 million cells for H3K27ac or 4 million cells for ΔNp63α using the iDeal ChIP-seq kit for TFs (Diagenode: C01010055). ChIP for ΔNp63α was carried out using ~ 3 ug each of 4A4 mouse monoclonal anti-ΔNp63 antibody or anti-HA antibody. ChIP for histone mark H3K27ac was performed using ~ 2 ug of H3K27ac (Diagenode: C15410174) antibody. Sequencing libraries were prepared using ThruPLEX DNA-seq kit from Rubicon Genomics. Samples were submitted to University at Buffalo Genomics and Bioinformatics Core (University at Buffalo, State University of New York; Buffalo, New York) and sequenced on a HiSeq using Standard 50-Cycle Single Read Sequencing. Sequencing and quality control were also performed at the University at Buffalo Genomics and Bioinformatics Core.

### Data analysis

Raw sequencing reads from K562 (4A4, HA, H3K27ac and inputs) and HepG2 (HA and input) were analyzed through an identical pipeline as performed before [20]. ChIP-seq experiments on the non-mutated p63 exceeded ENCODE quality standards [49]. ChIP-seq experiments for ΔNp63α(R304W) failed ENCODE quality standards as expected from a protein that does not bind to genomic DNA. Datasets were aligned to either hg19 or hg38 dependent on the downstream data analysis. All available Histone ChIP-seq and DNase-seq conducted in K562 and HepG2 were downloaded from the ENCODE repository: http://genome.ucsc.edu/ENCODE/. Chromatin State Segmentation files K562ChromHMM, NHEKChromHMM and HepG2ChromHMM were downloaded from GEO GSM936088, GEO GSM936087, and GEO GSM936090. K562 and HepG2 DNaseI/FAIRE/ChIP Synthesis file was downloaded from GEO GSM1002657, and GEO GSM1002654. MNase-seq data were from Mieczkowski et al. 2016 sample GSM2083140 [50], and analyzed as previously described [51, 52]. Additional analysis of MNase-seq datasets from [53] was performed using the NucMap database [54] using nucleosome positions determined with DANPOS or iNPS [55, 56]. Symmetry of nucleosome occupancy at p63BS was determined with ArchAlign with 0-bp shifts and region reversal enabled [23]. ChIP-seq binding sites annotation was done using annotatePeaks.pl from the HOMER package [57].

### Identifying the chromatin profile at p63 bound sites

Histone mark ChIP-seq data in K562, NHEK, and HepG2 cell lines were obtained from ENCODE consortium [21]. The coordinates for p63 bound sites in NHEK were obtained from previous study [20]. Using ArchTex histone modifications and MNase-seq data were plotted for a 2-kb window at 10-bp resolution with a standardized tag count of 100 million or 1 billion, respectively [58]. This analysis was performed in each 3 cell lines, respectively. An average signal across a 2-kb window centered at the ΔNp63α binding site was plotted for corresponding histone marks described in the main text in 3 different cells, respectively. k-means clustering algorithm implemented in Cluster 3.0 software was applied [59]. The heatmap was generated via Java TreeView software [60].

### p63–nucleosome in vitro binding assay

Expression and purification of His-tagged ΔNp63γ protein were performed as described previously [61]. Protein–nucleosome binding assays were carried out in duplicate with purified nucleosomes and ΔNp63γ protein [24]. Two p63BS were used, an adapted high-affinity ideal sequence: 5′-GGGCATGTCCGGGCATGTCC-3′ [62, 63] and a natural intermediate-affinity sequence from the *CDKN1A* promoter: 5′-AGACTGGGCATGTCTGGGCA-3′ [64]. 14 nucleosome sequences were designed starting from the 217-bp Widom 601 sequence and compared to non-specific binding to 2 control sequences (Additional file 2: Table S1). Protein binding was detected by mobility shift assay on 4% (w/v) native polyacrylamide gels. The original 601 sequence contains a TP63 half-site core (CATG) located just outside the nucleosome edge. To ensure that this sequence does not affect the binding assays we modified this sequence to AGGT. We called it ‘601-modified’, which was regarded as an additional control sequence. The original Widom 601 DNA (601) was still used in the study and had indistinguishable results compare to ‘601-modified’. All visual bands were excised from the gel, as well as the bands at the same locations in the other lanes. DNA from each band was extracted, purified, and then quantified by qPCR. All samples were multiplexed and sequenced on a MiSeq using 2 × 150-bp paired-end sequencing. Sequencing was performed at the University at Buffalo Genomics and Bioinformatics Core. Quality sequence reads were mapped to each specific starting sequence using Blat [65]. The results were then analyzed relative to control/non-specific binding (relative shift). Relative shift is determined from the non-shifted nucleosome bands and controls technical variability introduced by gel-excision, PCR, NGS-library construction, or NGS sequencing. In this method each specific nucleosome sequence is measured relative to non-specific binding (control 601 fragment):

$${\text{Relative}}\;{\text{shift}} = - \log _{2} \left( {{\raise0.7ex\hbox{${\frac{{{\text{reads}}\;{\text{nuclesome}}_{N} }}{{{\text{reads}}\;{\text{nucleosome}}_{{601}} }}}$} \!\mathord{\left/ {\vphantom {{\frac{{{\text{reads}}\;{\text{nuclesome}}_{N} }}{{{\text{reads}}\;{\text{nucleosome}}_{{601}} }}} {\frac{{{\text{reads}}\;{\text{nucleosome}}\;{\text{input}}_{N} }}{{{\text{reads}}\;{\text{nucleosome}}\;{\text{input}}_{{601}} }}}}}\right.\kern-\nulldelimiterspace} \!\lower0.7ex\hbox{${\frac{{{\text{reads}}\;{\text{nucleosome}}\;{\text{input}}_{N} }}{{{\text{reads}}\;{\text{nucleosome}}\;{\text{input}}_{{601}} }}}$}}} \right),$$

where *N* is one of the 16 nucleosome sequences, 601 is the control nucleosome sequence, reads nucleosome is the nucleosome band at specific concentration of ΔNp63, reads nucleosome input is the nucleosome band in input lane without any ΔNp63 added.

## Supplementary Information

**Additional file 1.** Figures and corresponding legends for supplementary figures 1–8.

**Additional file 2: Table S1.** Sequence details for competitive nucleosome binding assays.

## Data Availability

ChIP-seq datasets are available at NCBI GEO GSE140329 and in vitro nucleosome binding data available at NCBI SRA PRJNA588790.
