# Supplementary material for: ΔNp63 is a pioneer factor that binds inaccessible chromatin and elicits chromatin remodeling
Source: Epigenetics Chromatin. 2021 Apr 17;14:20. doi: 10.1186/s13072-021-00394-8 (PMC8053304; doi:10.1186/s13072-021-00394-8)
Supplement: Supplementary file 1 — Additional file 1. Figures and corresponding legends for supplementary figures 1–8. [file 13072_2021_394_MOESM1_ESM.pdf]

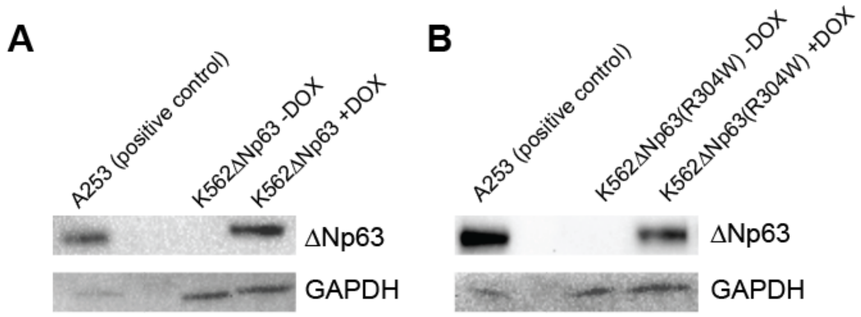

Figure S1. Western blot analysis confirmed the expression of ΔNp63 in K562.

(A) ΔNp63α expression was induced in K562 with 300ng/ml of doxycycline (DOX). A253 is an epithelial cell line originally expressing ΔNp63, which was used as positive control here. (B) ΔNp63(R304W) expression after Dox induction in K562.

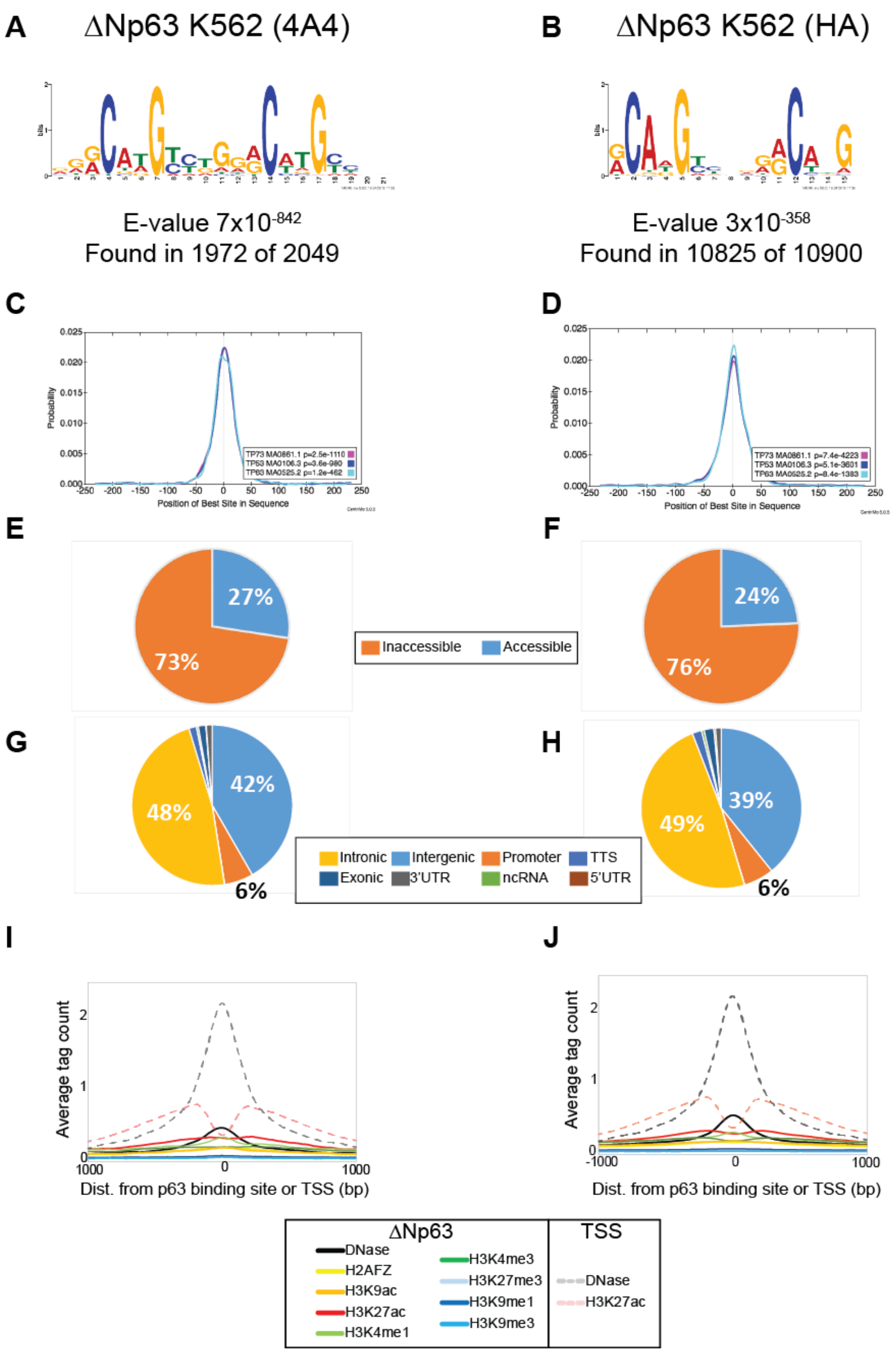

Figure S2. Replicate ΔNp63 ChIP-seq experiments.

(A-B) MEME analysis for ΔNp63 ChIP-seq replicates 1 and 2 [4]. **(C-D)** CentriMo analysis showing the center enrichment for three p53 family members' motif (p53, p63, p73) for ΔNp63 ChIP-seq replicates 1 and 2 [7]. **(E-F)** Chromatin accessibility ΔNp63 bound sites for each replicate in K562. Accessibility is defined from the synthesis track of DNase and FAIRE from ENCODE (GEO GSE40833 GSM1002657) [8]. (**G-H**) Genomic annotation for ΔNp63 bound sites for each replicate in K562. (**I-J**) Average chromatin architecture in K562 at ΔNp63 ChIP-seq summits for each replicate experiment compared to 72291 transcriptional start site (TSS). Data is plotted for 2 kb flanking the summit or TSS.

A

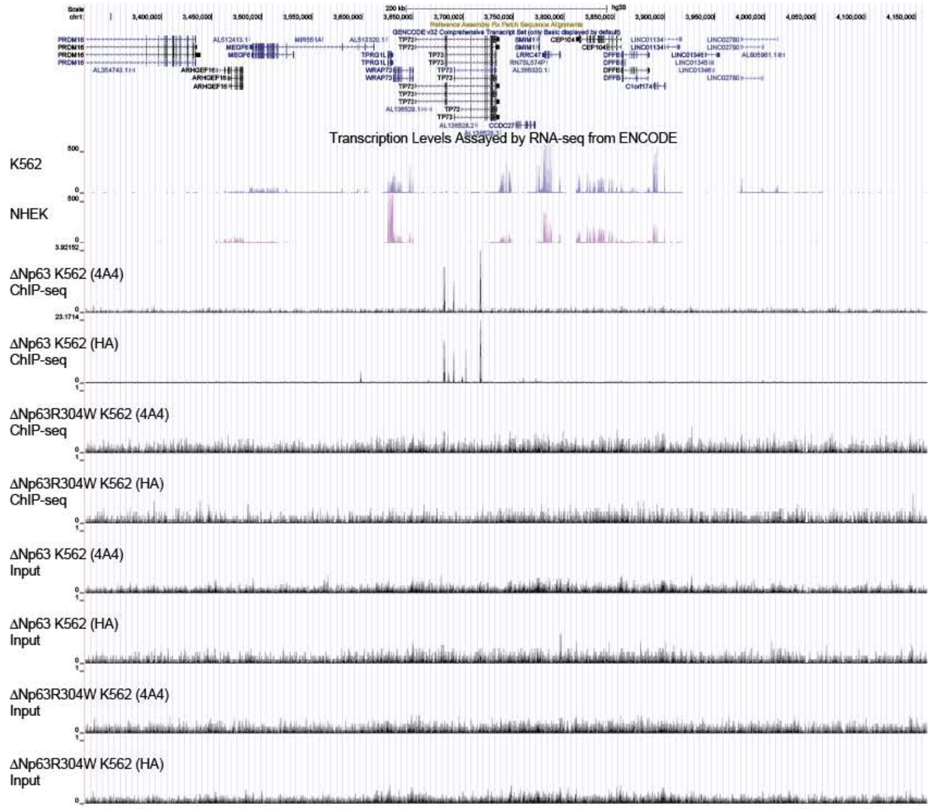
Transcription Levels Assayed by RNA-seq from ENCODE
K562
NHEK
ΔNp63 K562 (4A4)
ChIP-seq
ΔNp63 K562 (HA)
ChIP-seq
ΔNp63R304W K562 (4A4)
ChIP-seq
ΔNp63R304W K562 (HA)
ChIP-seq
ΔNp63 K562 (4A4)
Input
ΔNp63 K562 (HA)
Input
ΔNp63R304W K562 (4A4)
Input
ΔNp63R304W K562 (HA)
Input

B

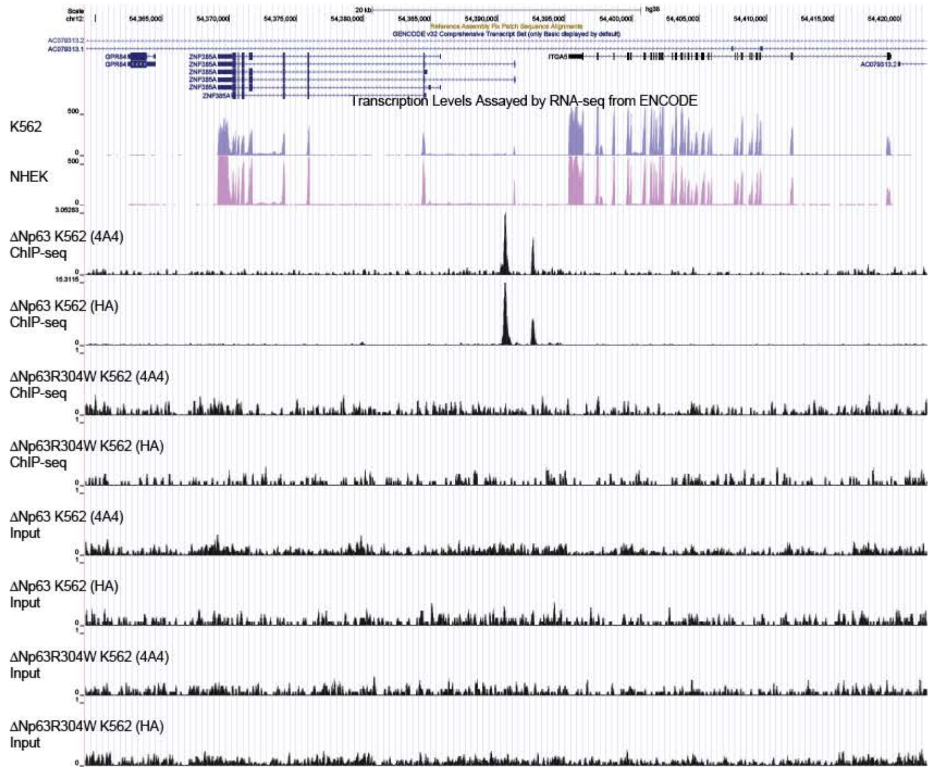
Transcription Levels Assayed by RNA-seq from ENCODE
K562
NHEK
ΔNp63 K562 (4A4)
ChIP-seq
ΔNp63 K562 (HA)
ChIP-seq
ΔNp63R304W K562 (4A4)
ChIP-seq
ΔNp63R304W K562 (HA)
ChIP-seq
ΔNp63 K562 (4A4)
Input
ΔNp63 K562 (HA)
Input
ΔNp63R304W K562 (4A4)
Input
ΔNp63R304W K562 (HA)
Input

C

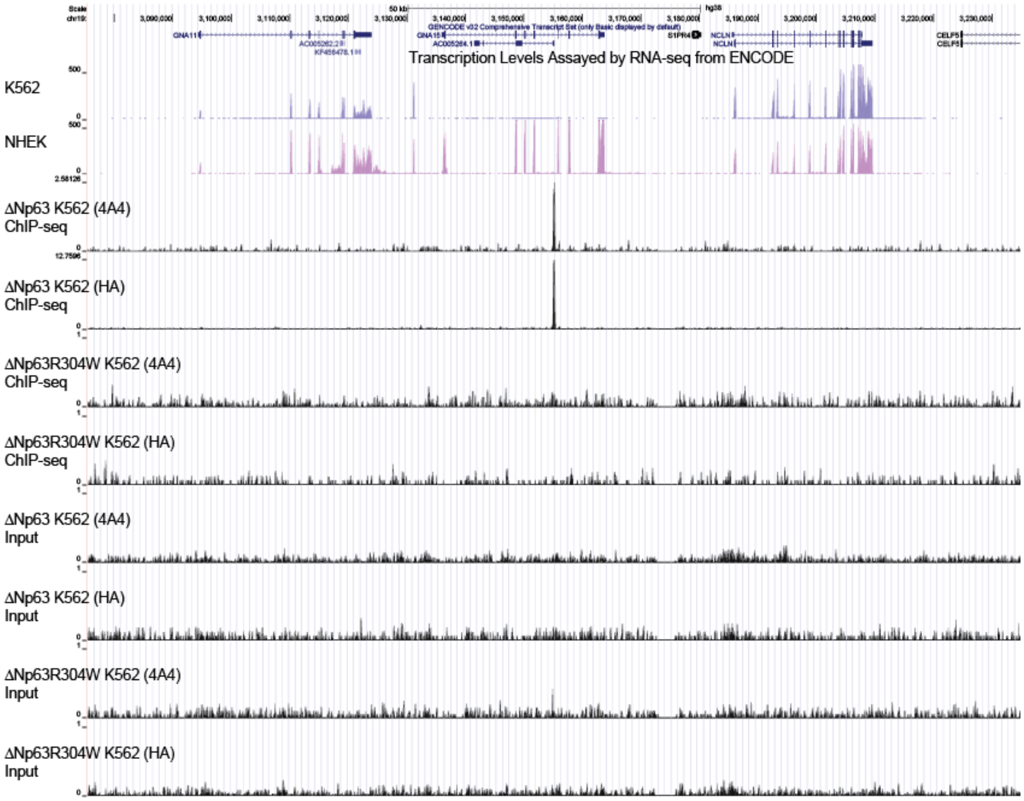

D

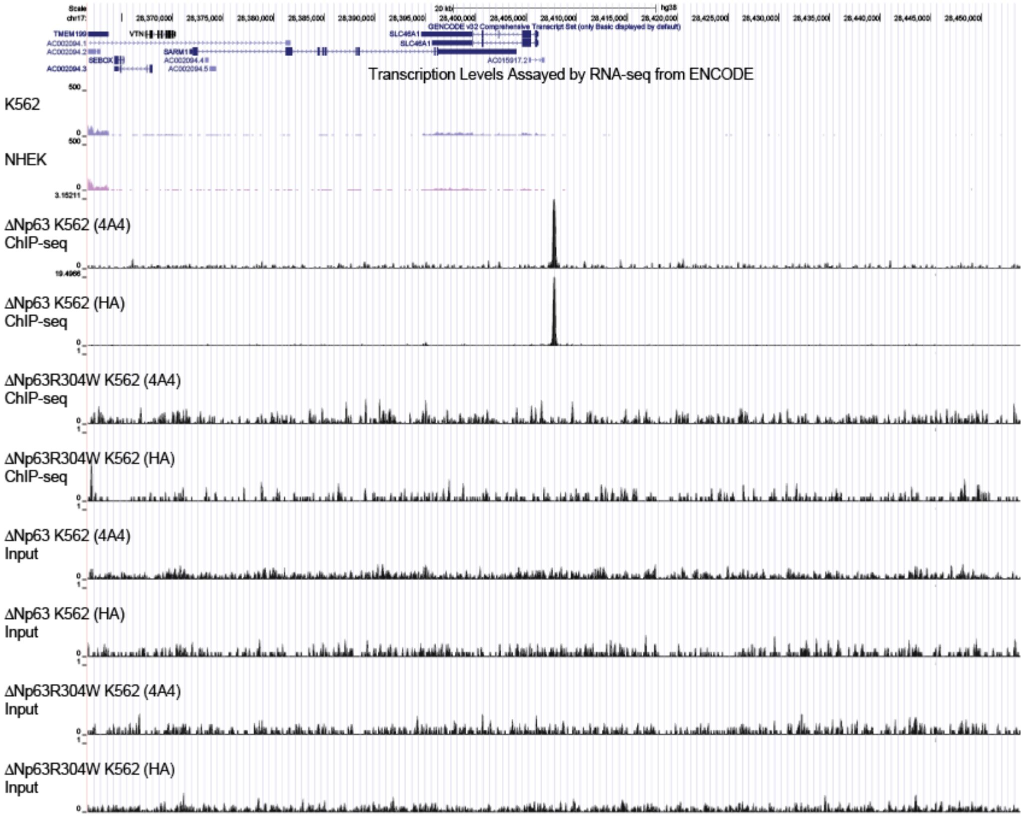

Figure S3. Browser screen shots showing ChIP-seq experiments. Replicate ChIP-seq experiments using antibodies 4A4 and HA for ΔNp63α and ΔNp63(R304W) with their accompanying inputs. Transcription levels from ENCODE polyadenylated RNA-seq is shown for K562 and NHEK cell lines.

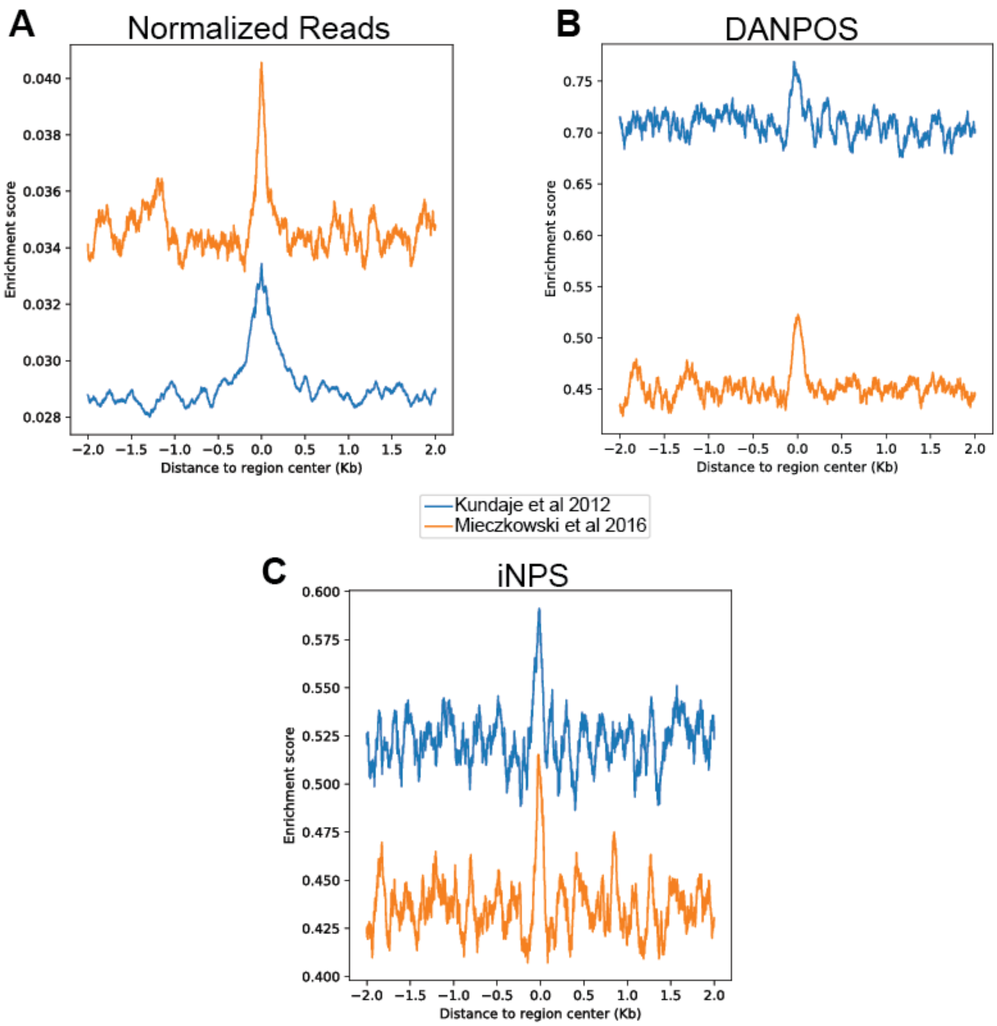

Figure S4. Nucleosomes are enriched at ΔNp63α binding sites in K562. Two K562 datasets from the NucMap database from Kundaje et al 2012 and Mieczkowski 2016 are shown [1-3]. (**A**) Normalized reads, (**B)** called nucleosomes with DANPOS [5], and (C) called nucleosomes with iNPS [6].

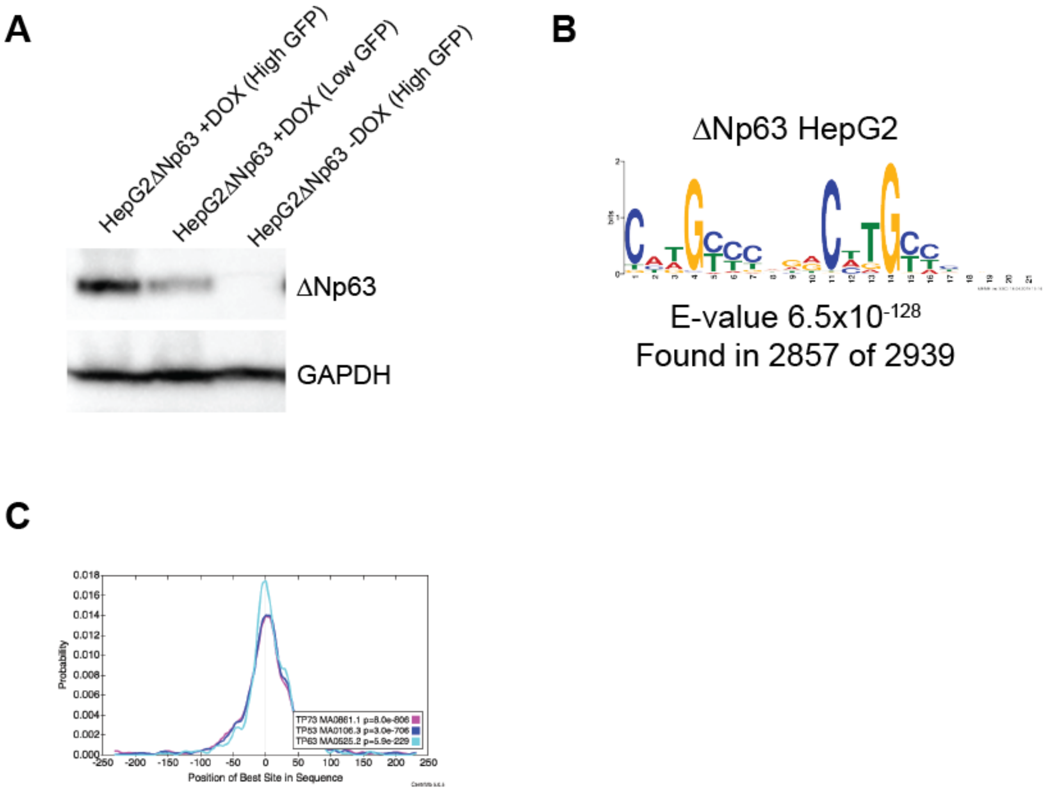

Figure S5. **Ectopic expression of p63 protein in HepG2.**

(**A**) Western blot analysis of ΔNp63α expression induced in HepG2 cells with 300ng/ml of doxycycline (DOX). (**B**) MEME analysis of ΔNp63 ChIP-seq from HepG2 [4]. (C) CentriMo analysis showing the center enrichment for three p53 family members' motif (p53, p63, p73) for HepG2 ΔNp63 ChIP-seq [7].

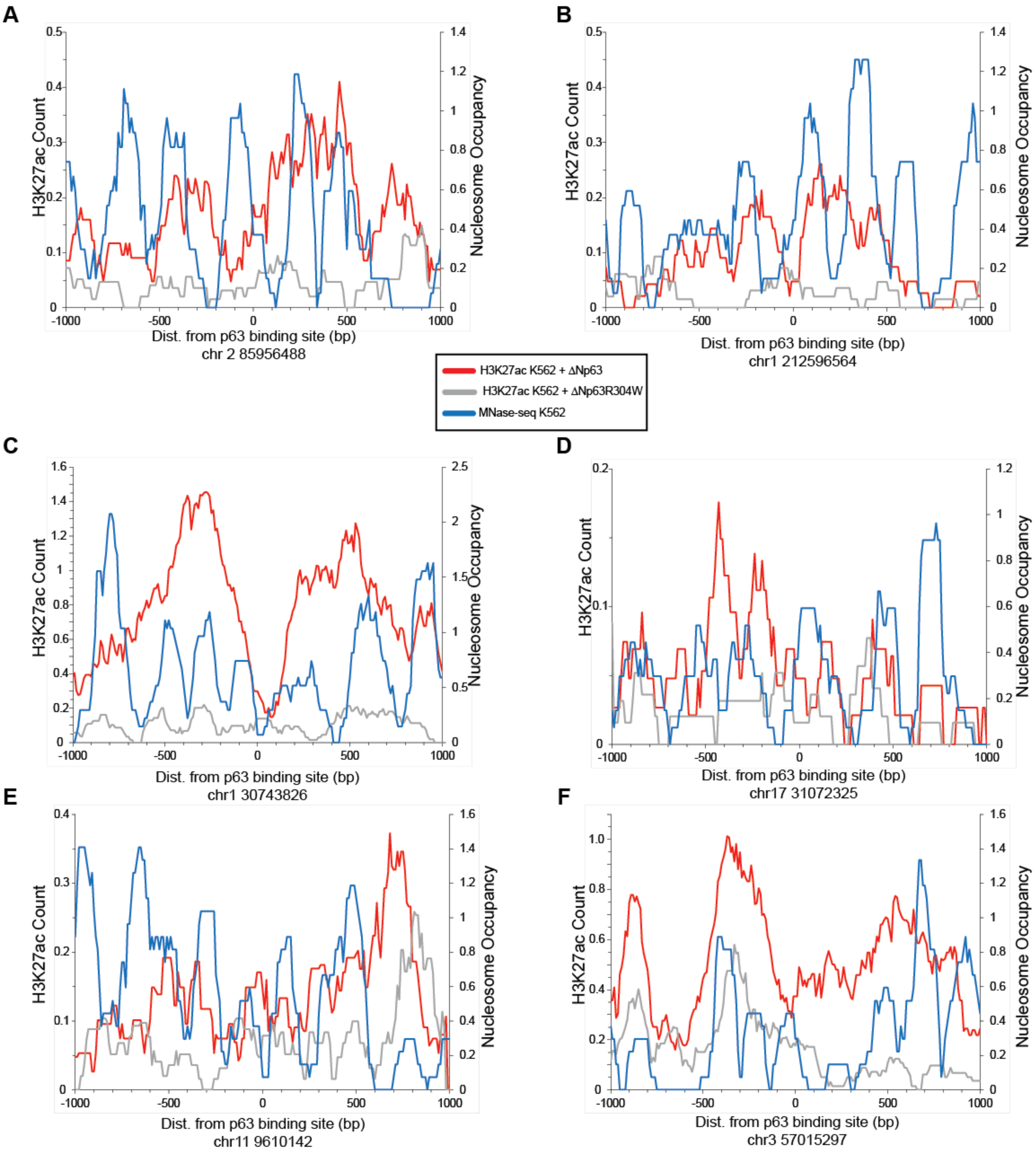

Figure S7. Nucleosome occupancy and H3K27ac at ΔNp63 binding sites. (A-F) H3K27ac at two p63BS. Average H3K27ac from two replicate experiments for K562 + ΔNp63 and K562 + ΔNp63(R304W). Nucleosome occupancy as determined from MNase-seq from non-p63 expressing K562 [3].

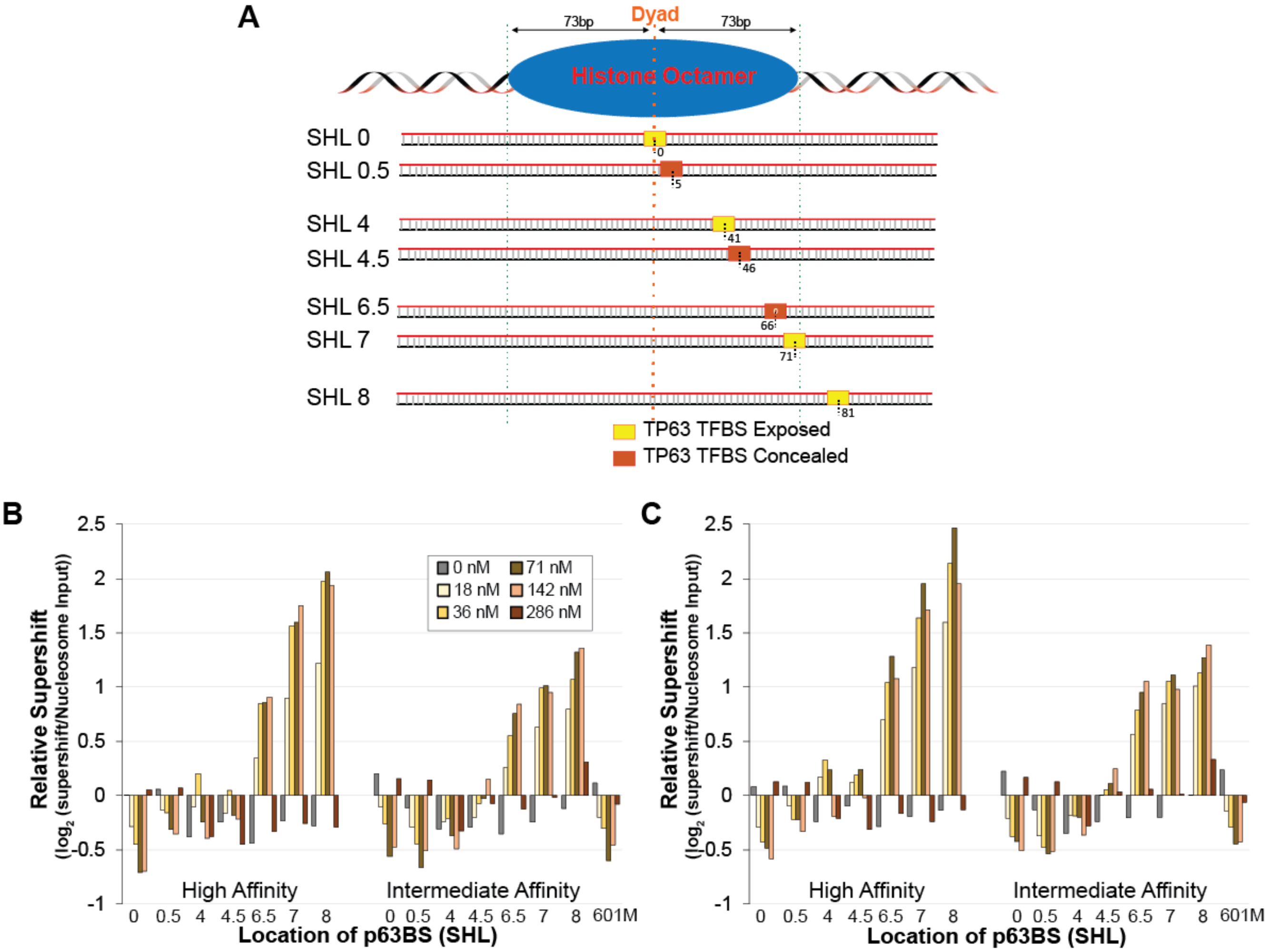

Figure S8. Relative supershift values from the supershifted bands (Ss).

(A) 217-bp dsDNA library was designed containing p63BS in various nucleosomal positions. A 20-bp-long p63BS (low- and high-affinity) was placed at different positions with 0-bp, 5-bp, 41-bp, 46-bp, 66-bp, 71-bp, 81-bp away from the dyad. The superhelix location (SHL) is designated for each nucleosome sequence. **(B-C)** Lanes contain the following:1– 0.25 pmol nucleosomes, 2-5 contain 0.25 pmol of nucleosomes with 18, 36, 71, 142, or 286 nM of p63 (0.125, 0.25, 0.5, 1, or 2 pmol).

1. Zhao Y, Wang J, Liang F, Liu Y, Wang Q, Zhang H, Jiang M, Zhang Z, Zhao W, Bao Y *et al*: **NucMap: a database of genome-wide nucleosome positioning map across species**. *Nucleic Acids Res* 2019, **47**(D1):D163-D169.
2. Kundaje A, Kyriazopoulou-Panagiotopoulou S, Libbrecht M, Smith CL, Raha D, Winters EE, Johnson SM, Snyder M, Batzoglou S, Sidow A: **Ubiquitous heterogeneity and asymmetry of the chromatin environment at regulatory elements**. *Genome Res* 2012, **22**(9):1735-1747.
3. Mieczkowski J, Cook A, Bowman SK, Mueller B, Alver BH, Kundu S, Deaton AM, Urban JA, Larschan E, Park PJ *et al*: **MNase titration reveals differences between nucleosome occupancy and chromatin accessibility**. *Nat Commun* 2016, **7**:11485.
4. Bailey TL, Boden M, Buske FA, Frith M, Grant CE, Clementi L, Ren J, Li WW, Noble WS: **MEME SUITE: tools for motif discovery and searching**. *Nucleic Acids Res* 2009, **37**(Web Server issue):W202-208.
5. Chen K, Xi Y, Pan X, Li Z, Kaestner K, Tyler J, Dent S, He X, Li W: **DANPOS: dynamic analysis of nucleosome position and occupancy by sequencing**. *Genome Res* 2013, **23**(2):341-351.
6. Chen W, Liu Y, Zhu S, Green CD, Wei G, Han JD: **Improved nucleosome-positioning algorithm iNPS for accurate nucleosome positioning from sequencing data**. *Nat Commun* 2014, **5**:4909.
7. Bailey TL, Machanick P: **Inferring direct DNA binding from ChIP-seq**. *Nucleic Acids Res* 2012, **40**(17):e128.
8. Consortium EP: **An integrated encyclopedia of DNA elements in the human genome**. *Nature* 2012, **489**(7414):57-74.
