# Supplementary material for: ΔNp63 is a pioneer factor that binds inaccessible chromatin and elicits chromatin remodeling
Source: Epigenetics Chromatin. 2021 Apr 17;14:20. doi: 10.1186/s13072-021-00394-8 (PMC8053304; doi:10.1186/s13072-021-00394-8)
Supplement: Supplementary file 2 — Additional file 2: Table S1. Sequence details for competitive nucleosome binding assays. [file 13072_2021_394_MOESM2_ESM.pdf]

Table S1. Sequence details of competitive nucleosome binding assays

Binding site location	p63 binding site	Nucleosome Sequence
SHL 0	High Affinity	GATGGACCCTATACGCGGCCGCCCTGGAGAATCCCGGTGCCGAGGCCGCTCAATTGGTCGTAGACAGCTCTAGCACCGCTTAAACGCACGTGGGCATGTCCGGGCATGTCCTAACCG<br>CCAAGGGGATTACTCCCTAGTCTCCAGGCACGTGTCAGATATATACATCCTGTGAGGTTATTGAACAGCGACCTTGCCGGTGCCAGTCGGATAGTGTTCC
SHL 0.5	High Affinity	GATGGACCCTATACGCGGCCGCCCTGGAGAATCCCGGTGCCGAGGCCGCTCAATTGGTCGTAGACAGCTCTAGCACCGCTTAAACGCACGTACGCGGGGCATGTCCGGGCATGTCCG<br>CCAAGGGGATTACTCCCTAGTCTCCAGGCACGTGTCAGATATATACATCCTGTGAGGTTATTGAACAGCGACCTTGCCGGTGCCAGTCGGATAGTGTTCC
SHL 4	High Affinity	GATGGACCCTATACGCGGCCGCCCTGGAGAATCCCGGTGCCGAGGCCGCTCAATTGGTCGTAGACAGCTCTAGCACCGCTTAAACGCACGTACGCG**C**TGTCCCCCGCGTTTTAACCG<br>CCAAGGGGATTACTCGGGCATGTCCGGGCATGTCCCAGATATATACATCCTGTGAGGTTATTGAACAGCGACCTTGCCGGTGCCAGTCGGATAGTGTTCC
SHL 4.5	High Affinity	GATGGACCCTATACGCGGCCGCCCTGGAGAATCCCGGTGCCGAGGCCGCTCAATTGGTCGTAGACAGCTCTAGCACCGCTTAAACGCACGTACGCG**C**TGTCCCCCGCGTTTTAACCG<br>CCAAGGGGATTACTCCCTAGGGGCATGTCCGGGCATGTCCATATACATCCTGTGAGGTTATTGAACAGCGACCTTGCCGGTGCCAGTCGGATAGTGTTCC
SHL 7	High Affinity	GATGGACCCTATACGCGGCCGCCCTGGAGAATCCCGGTGCCGAGGCCGCTCAATTGGTCGTAGACAGCTCTAGCACCGCTTAAACGCACGTACGCG**C**TGTCCCCCGCGTTTTAACCG<br>CCAAGGGGATTACTCCCTAGTCTCCAGGCACGTGTCAGATATATAGGGCATGTCCGGGCATGTCCCAGCGACCTTGCCGGTGCCAGTCGGATAGTGTTCC
SHL 6.5	High Affinity	GATGGACCCTATACGCGGCCGCCCTGGAGAATCCCGGTGCCGAGGCCGCTCAATTGGTCGTAGACAGCTCTAGCACCGCTTAAACGCACGTACGCG**C**TGTCCCCCGCGTTTTAACCG<br>CCAAGGGGATTACTCCCTAGTCTCCAGGCACGTGTCAGATGGGCATGTCCGGGCATGTCCTTGAACAGCGACCTTGCCGGTGCCAGTCGGATAGTGTTCC
SHL8	High Affinity	GATGGACCCTATACGCGGCCGCCCTGGAGAATCCCGGTGCCGAGGCCGCTCAATTGGTCGTAGACAGCTCTAGCACCGCTTAAACGCACGTACGCG**C**TGTCCCCCGCGTTTTAACCG<br>CCAAGGGGATTACTCCCTAGTCTCCAGGCACGTGTCAGATATATACATCCTGTGCGGGCATGTCCGGGCATGTCCGCCGGTGCCAGTCGGATAGTGTTCC
SHL 0	Intermediate Affinity	GATGGACCCTATACGCGGCCGCCCTGGAGAATCCCGGTGCCGAGGCCGCTCAATTGGTCGTAGACAGCTCTAGCACCGCTTAAACGCACGTAGACT**G**GGCATGTCTGGGCATAACCG<br>CCAAGGGGATTACTCCCTAGTCTCCAGGCACGTGTCAGATATATACATCCTGTGCATGTATTGAACAGCGACCTTGCCGGTGCCAGTCGGATAGTGTTCC
SHL 0.5	Intermediate Affinity	GATGGACCCTATACGCGGCCGCCCTGGAGAATCCCGGTGCCGAGGCCGCTCAATTGGTCGTAGACAGCTCTAGCACCGCTTAAACGCACGTACGCG**A**GACTGGGCATGTCTGGGCAG<br>CCAAGGGGATTACTCCCTAGTCTCCAGGCACGTGTCAGATATATACATCCTGTGCATGTATTGAACAGCGACCTTGCCGGTGCCAGTCGGATAGTGTTCC
SHL 4	Intermediate Affinity	GATGGACCCTATACGCGGCCGCCCTGGAGAATCCCGGTGCCGAGGCCGCTCAATTGGTCGTAGACAGCTCTAGCACCGCTTAAACGCACGTACGCG**C**TGTCCCCCGCGTTTTAACCG<br>CCAAGGGGATTACTCAGACTGGGCATGTCTGGGCACAGATATATACATCCTGTGCATGTATTGAACAGCGACCTTGCCGGTGCCAGTCGGATAGTGTTCC
SHL 4.5	Intermediate Affinity	GATGGACCCTATACGCGGCCGCCCTGGAGAATCCCGGTGCCGAGGCCGCTCAATTGGTCGTAGACAGCTCTAGCACCGCTTAAACGCACGTACGCG**C**TGTCCCCCGCGTTTTAACCG<br>CCAAGGGGATTACTCCCTAGAGACTGGGCATGTCTGGGCAATATACATCCTGTGCATGTATTGAACAGCGACCTTGCCGGTGCCAGTCGGATAGTGTTCC
SHL 7	Intermediate Affinity	GATGGACCCTATACGCGGCCGCCCTGGAGAATCCCGGTGCCGAGGCCGCTCAATTGGTCGTAGACAGCTCTAGCACCGCTTAAACGCACGTACGCG**C**TGTCCCCCGCGTTTTAACCG<br>CCAAGGGGATTACTCCCTAGTCTCCAGGCACGTGTCAGATATATAAGACTGGGCATGTCTGGGCACAGCGACCTTGCCGGTGCCAGTCGGATAGTGTTCC
SHL 6.5	Intermediate Affinity	GATGGACCCTATACGCGGCCGCCCTGGAGAATCCCGGTGCCGAGGCCGCTCAATTGGTCGTAGACAGCTCTAGCACCGCTTAAACGCACGTACGCG**C**TGTCCCCCGCGTTTTAACCG<br>CCAAGGGGATTACTCCCTAGTCTCCAGGCACGTGTCAGATAGACTGGGCATGTCTGGGCATTGAACAGCGACCTTGCCGGTGCCAGTCGGATAGTGTTCC
SHL 8	Intermediate Affinity	GATGGACCCTATACGCGGCCGCCCTGGAGAATCCCGGTGCCGAGGCCGCTCAATTGGTCGTAGACAGCTCTAGCACCGCTTAAACGCACGTACGCG**C**TGTCCCCCGCGTTTTAACCG<br>CCAAGGGGATTACTCCCTAGTCTCCAGGCACGTGTCAGATATATACATCCTGTGCAGACTGGGCATGTCTGGGCAGCCGGTGCCAGTCGGATAGTGTTCC
601-Modified	Control	GATGGACCCTATACGCGGCCGCCCTGGAGAATCCCGGTGCCGAGGCCGCTCAATTGGTCGTAGACAGCTCTAGCACCGCTTAAACGCACGTACGCG**C**TGTCCCCCGCGTTTTAACCG<br>CCAAGGGGATTACTCCCTAGTCTCCAGGCACGTGTCAGATATATACATCCTGTGAGGTTATTGAACAGCGACCTTGCCGGTGCCAGTCGGATAGTGTTCC
601	Control	GATGGACCCTATACGCGGCCGCCCTGGAGAATCCCGGTGCCGAGGCCGCTCAATTGGTCGTAGACAGCTCTAGCACCGCTTAAACGCACGTACGCG**C**TGTCCCCCGCGTTTTAACCG<br>CCAAGGGGATTACTCCCTAGTCTCCAGGCACGTGTCAGATATATACATCCTGTGCATGTATTGAACAGCGACCTTGCCGGTGCCAGTCGGATAGTGTTCC
